# MCC950 Alleviates Fat Embolism-Induced Acute Respiratory Distress Syndrome Through Dual Modulation of NLRP3 Inflammasome and ERK Pathways

**DOI:** 10.3390/ijms26157571

**Published:** 2025-08-05

**Authors:** Chin-Kuo Lin, Zheng-Wei Chen, Yu-Hao Lin, Cheng-Ta Yang, Chung-Sheng Shi, Chieh-Mo Lin, Tzu Hsiung Huang, Justin Ching Hsien Lu, Kwok-Tung Lu, Yi-Ling Yang

**Affiliations:** 1 Division of Pulmonary Infection and Critical Care, Department of Pulmonary and Critical Care Medicine, Chang Gung Memorial Hospital, Chiayi 20401, Taiwan; lingh@cgmh.org.tw (C.-K.L.); f124510714@cgmh.org.tw (C.-M.L.); 2 Graduate Institute of Clinical Medicine Sciences, College of Medicine, Chang Gung University, Taoyuan 33302, Taiwan; csshi@mail.cgu.edu.tw (C.-S.S.); world@cgmh.org.tw (T.H.H.); 3 Department of Biochemical Science and Technology, National Chia-Yi University, Chia-Yi 60004, Taiwan; aaa2731249@gmail.com (Z.-W.C.); douglas4387863@gmail.com (Y.-H.L.); 4 Department of Pulmonary and Critical Care Medicine, Chang Gung Memorial Hospital, Taoyuan 33305, Taiwan; yang1946@cgmh.org.tw; 5 Department of Respiratory Care, College of Medicine, Chang Gung University, Taoyuan 33302, Taiwan; 6 Division of Colon and Rectal Surgery, Department of Surgery, Chang Gung Memorial Hospital, Chiayi 61363, Taiwan; 7 Department of Respiratory Therapy, Chang Gung Memorial Hospital, Chiayi 61363, Taiwan; 8 School of Medicine, Fu Jen Catholic University, New Taipei 242062, Taiwan; jasonlu1374@gmail.com; 9 Department of Life Science, National Taiwan Normal University, Taipei 10659, Taiwan

**Keywords:** fat embolism, acute respiratory distress syndrome (ARDS), NOD-like receptor pyrin domain-containing 3 (NLRP3), MCC950, reactive oxygen species (ROS)

## Abstract

Fat embolism is a critical medical emergency often resulting from long bone fractures or amputations, leading to acute respiratory distress syndrome (ARDS). The NOD-like receptor pyrin domain-containing 3 (NLRP3) inflammasome, a key regulator of innate immunity, is activated by reactive oxygen species and tissue damage, contributing to inflammatory responses. This study examines the role of NLRP3 in fat embolism-induced ARDS and evaluates the therapeutic potential of MCC950, a selective NLRP3 antagonist. Fat embolism was induced by fatty micelle injection into the tail vein of Sprague Dawley rats. Pulmonary injury was assessed through lung weight gain as an edema indicator, NLRP3 expression via Western blot, and IL-1β levels using ELISA. Histological damage and macrophage infiltration were evaluated with hematoxylin and eosin staining. Fat embolism significantly increased pulmonary NLRP3 expression, lipid peroxidation, IL-1β release, and macrophage infiltration within four hours, accompanied by severe pulmonary edema. NLRP3 was localized in type I alveolar cells, co-localizing with aquaporin 5. Administration of MCC950 significantly reduced inflammatory responses, lipid peroxidation, pulmonary edema, and histological damage, while attenuating MAPK cascade phosphorylation of ERK and Raf. These findings suggest that NLRP3 plays a critical role in fat embolism-induced acute respiratory distress syndrome, and its inhibition by MCC950 may offer a promising therapeutic approach.

## 1. Introduction

Fat embolism syndrome (FES), a potentially life-threatening condition characterized by circulating fat globules, primarily occurs following traumatic injuries such as long bone or pelvic fractures, or surgical procedures like liposuction. Younger populations are disproportionately affected due to greater exposure to high-velocity trauma [1]. With mortality rates ranging from 7% to 36%, FES poses a significant clinical challenge [2]. The pathophysiology of FES involves a complex cascade of events initiated when fat globules enter the venous circulation. These emboli first impact the pulmonary capillary bed before potentially affecting other organ systems, including neurological, renal, hepatic, and cardiovascular systems [3,4]. Fat globules directly irritate the lung parenchyma, leading to increased alveolar dead space and hypoxia through compromised gas exchange, arising from pulmonary hyperpermeability, edema, and hemorrhage [1]. This pathological sequence frequently progresses to acute respiratory distress syndrome (ARDS).

Recent evidence has revealed that free fatty acids can trigger inflammasome activation through reactive oxygen species (ROS) generation and autophagy disruption, culminating in mature IL-1β secretion [5,6]. The NOD-like receptor pyrin domain-containing 3 (NLRP3) inflammasome has emerged as a central regulator of inflammatory responses, particularly in conditions involving lipid-mediated inflammation [6,7]. Predominantly expressed in immune and inflammatory cells, particularly macrophages, monocytes, and splenic neutrophils [8], NLRP3 mediates caspase-1 activation and IL-1β/IL-18 production. Various stimuli, including mitochondrial damage, ATP, K+ efflux, and Ca2+ influx from tissue damage, can trigger NLRP3 activation [9,10]. Notably, ROS generation, especially from mitochondria, is both an upstream trigger and downstream consequence of NLRP3 inflammasome activation, creating a potential feedback loop that amplifies inflammatory responses. This ROS-NLRP3 interaction is evidenced by the effectiveness of ROS inhibitors in preventing NLRP3 activation [11]. In addition, the p38δ-MAPK pathway is activated by intracellular stress signals triggered during ATP- and cholesterol crystal-induced NLRP3 inflammasome activation and is necessary for NLRP3-mediated IL-1β secretion [12].

NLRP3 Inflammasome activation has been implicated in various forms of acute lung injury and ARDS [13,14,15,16,17]. However, its specific role in FES-induced ARDS remains unclear. Given that free fatty acids are potent activators of NLRP3 [5,18] and that lipid-mediated inflammation is central to FES pathogenesis, we hypothesized that NLRP3 inflammasome activation might play a critical role in the development and progression of FES-induced ARDS. Therefore, this study aims to investigate the role of NLRP3 inflammasome in FES-induced ARDS by determining whether NLRP3 is essential for the development of FES-induced ARDS and to elucidate the underlying molecular mechanisms, potentially identifying new therapeutic targets for ARDS.

## 2. Results

### 2.1. NLRP3 Mediates Fat Embolism-Induced Acute Lung Injury

**Figure 1:**
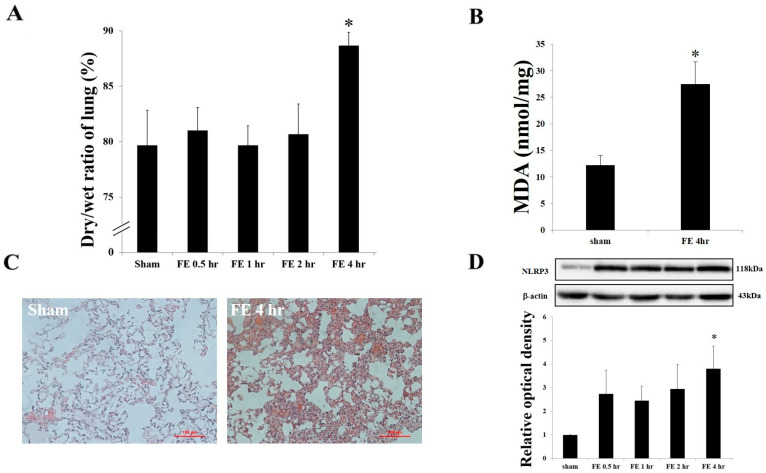
Fat embolism induces pulmonary damage and NLRP3 expression: (**A**) Quantification of lung weight gain (LWG) as a measure of pulmonary edema at 4 h post-fat embolism (FE) induction compared with sham controls. Data are presented as mean ± SEM (n = 6 per group). * *p* < 0.05 vs. sham group. (**B**) Measurement of lipid peroxidation levels using MDA assay in lung tissue 4 h after FE induction. Values are expressed as mean ± SEM (n = 6 per group). * *p* < 0.05 vs. sham group. (**C**) Representative hematoxylin and eosin (H&E) staining of lung sections showing alveolar damage 4 h after FE induction. Scale bar = 100 μm. (**D**) Western blot analysis and quantification of NLRP3 protein expression in lung tissue 4 h after FE induction. β-actin served as loading control. Data represent mean ± SEM (n = 5 per group). * *p* < 0.05 vs. sham group.

The temporal progression of fat embolism (FE)-induced lung edema was assessed by lung weight gain (LWG). Compared with the sham group (without FE induction), animals subjected to FE exhibited pronounced pulmonary edema at 4 h post-FE, with water content increasing significantly from 79.67% ± 3.18% to 88.67% ± 1.21% (Figure 1A). Lipid peroxidation, a key factor in FE-induced pulmonary damage, was assessed by the MDA and TBA colorimetric reaction. Results show significantly higher lipid peroxidation levels at 4 h post-FE induction compared with the sham group (Figure 1B). Alveolar damage, evaluated by H&E staining, also revealed marked pulmonary damage 4 h after FE treatment (Figure 1C), consistent with the results of pulmonary edema and the MDA assay. Pulmonary NLRP3 expression was significantly elevated at 4 h post-FE (Figure 1D), suggesting NLRP3′s potential involvement in FE-induced lung edema and pulmonary damage.

### 2.2. NLRP3 Inhibition by MCC950 Attenuates FE-Induced Lung Injury

**Figure 2:**
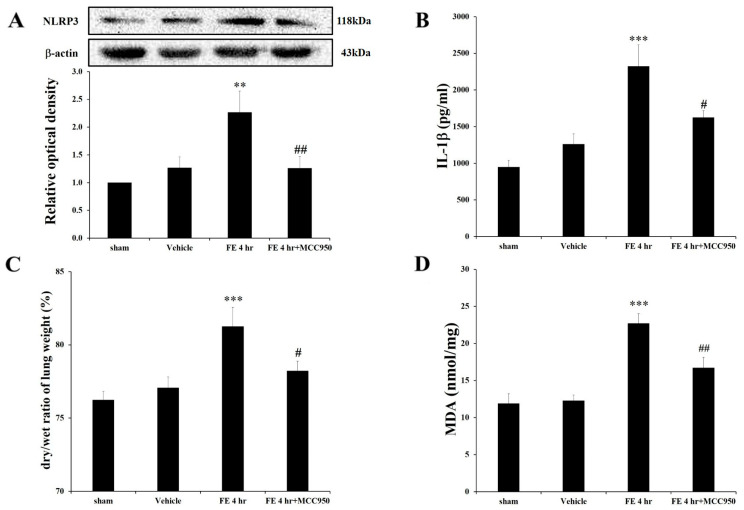
MCC950 attenuates FE-induced NLRP3 activation and pulmonary inflammation: (**A**) Effects of MCC950 (10 mg/kg) on FE-induced NLRP3 expression by Western blot analysis. Data are presented as mean ± SEM (n = 6 per group). (**B**) ELISA quantification of IL-1β levels in lung tissue after FE induction with or without MCC950 (10 mg/kg) treatment. Data are presented as mean ± SEM (n = 5 per group). (**C**) Effect of MCC950 (10 mg/kg) on FE-induced pulmonary edema measured by lung weight gain. Data are presented as mean ± SEM (n = 6 per group). (**D**) MDA levels in lung tissue comparing control, FE, and FE+MCC950 groups. Data are presented as mean ± SEM (n = 5 per group). ** *p* < 0.01, *** *p* < 0.001 were considered significantly different from sham values; # *p* < 0.05, ## *p* < 0.01 were considered significantly different from rats with FE treatment by the Mann–Whitney U-test.

To explore NLRP3′s involvement in fat embolism (FE)-induced edema, we administered MCC950 based on dose selection from previous studies. Our pilot study tested three doses of MCC950 on FE-induced NLRP3 expression. The 5 mg/kg dose showed no inhibitory effect, whereas the 10 mg/kg and 20 mg/kg doses significantly attenuated NLRP3 expression. To minimize non-specific effects, we selected the 10 mg/kg dose for subsequent experiments (Figure 2A).

We also assessed the expression of IL-1β, a key downstream inflammatory signal of NLRP3. ELISA analysis quantified IL-1β protein levels in the lavage fluid of left lung tissue after FE induction and MCC950 treatment. The results show a significant increase in pulmonary IL-1β expression 4 h after FE, which was significantly mitigated by MCC950 administration (Figure 2B). These findings suggest that MCC950 effectively suppresses inflammation, validating our dose selection.

**Figure 3:**
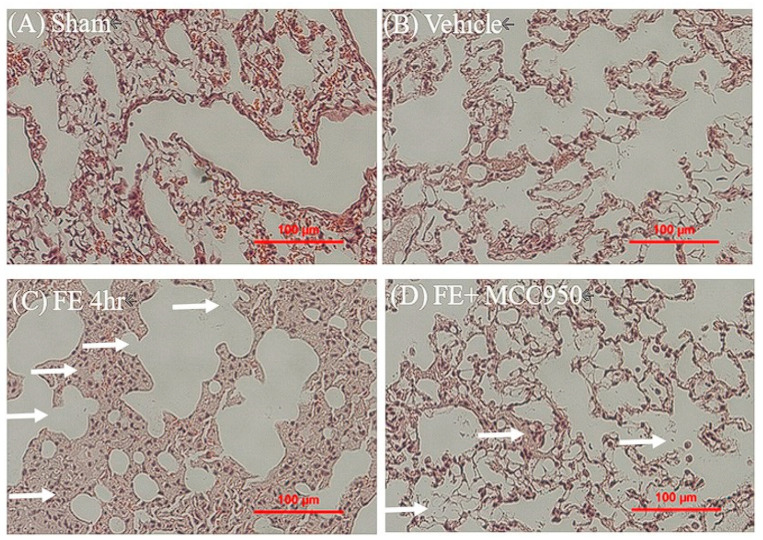
Histological analysis of MCC950′s protective effects against FE-induced lung injury. Representative H&E staining of lung sections from (**A**) control, (**B**) vehicle, (**C**) FE-induced, and (**D**) FE+MCC950-treated groups. White arrows indicate alveolar damage and inflammatory cell infiltration. Scale bar = 100 μm.

Compared with the FE-alone group, intravenous injection of MCC950 (10 mg/kg, I.V.) significantly reduced FE-induced pulmonary edema from 81.12% ± 1.34% to 78.13% ± 0.65%, indicating that NLRP3 mediates FE-induced pulmonary edema (Figure 2C). Furthermore, the potential protective effect of MCC950 on FE-induced pulmonary damage was evaluated using the MDA assay and hematoxylin and eosin staining. MCC950 administration led to a significant decrease in lipid peroxidation, evidenced by reduced MDA levels (Figure 2D). Moreover, MCC950 markedly reduced FE-induced pulmonary damage as evidenced by preserved alveolar structure and decreased inflammatory cell infiltration, with histological features resembling those of the control group (Figure 3). These findings further support the hypothesis that NLRP3 is crucial in FE-induced pulmonary edema and subsequent lung injury.

### 2.3. MCC950 Administration Also Inhibited the ERK and Raf Phosphorylation

**Figure 4:**
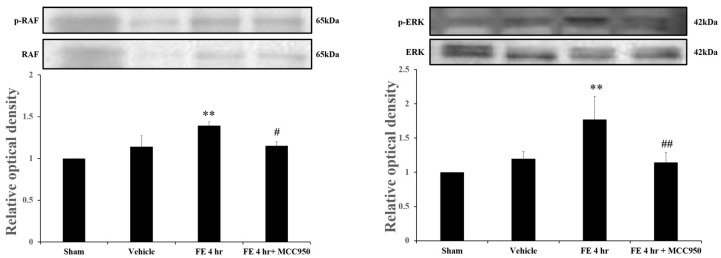
Effects of MCC950 on the pulmonary phosphorylation of Raf (p-Raf) and ERK (p-ERK) in sham rats (sham), rats with vehicle (vehicle), rats with fat embolism (FE), or rats treated with MCC950 (FE + MCC950). Upper: Western blot analysis of sham rats (sham), rats with vehicle (vehicle), rats with fat embolism (FE), or rats treated with MCC950 (10 mg/kg) (FE + MCC950). Lower: Relative density presented as folds compared with the sham group. Data are presented as the mean ± SEM values (n = 3). ** *p* < 0.01 were considered significantly different from sham values; and # *p*< 0.05, ## *p*< 0.01 were considered significantly different from rats with FE treatment by the Mann–Whitney U-test.

In our previous studies, we found that MAPK phosphorylation is critical to FE-induced pulmonary edema and damage. In the current study, we found that administration of MCC950 not only blocked the expression of NLRP3 but also inhibited the phosphorylation of ERK and Raf (Figure 4). Since the MAPK cascade has been shown to play an important role in FE-induced ARDS and pulmonary injury, these results suggest that MCC950 mitigates FE-induced pulmonary injury via inhibition of the ERK cascade.

### 2.4. Cellular Distribution of NLRP3 in FE-Induced Lung Injury

**Figure 5:**
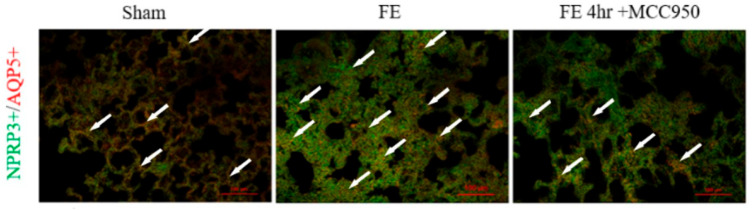
Cellular localization of NLRP3 in FE-induced lung injury and the effects of MCC950 administration: Double immunofluorescence staining showing co-localization of NLRP3 (green) with aquaporin-5 (red, type I alveolar cell marker). Merged images display overlapping regions, indicated by the white arrow. Scale bar = 100 μm. After FE, the expression of NLRP3 significantly increased and co-localized with aquaporin-5, and administration of MCC950 attenuated the expression of NLRP3 in type I alveolar cells.

To determine the cellular localization of NLRP3 in FE-induced lung injury, we performed immunofluorescence analysis using cell-type-specific markers. Double immunofluorescence staining revealed that FE-induced NLRP3 expression was predominantly localized to type I alveolar epithelial cells, as demonstrated by extensive co-localization with the type I cell marker Aquaporin-5 (red). The specificity of this localization was confirmed by minimal overlap with markers for type II alveolar cells and endothelial cells in our preliminary study. Compared with sham control animals, the NLRP3-positive type I cells numbers significantly increased in the FE-treated group (Figure 5); MCC950 treatment substantially reduced both the intensity and extent of NLRP3 immunoreactivity in type I cells (Figure 5), suggesting MCC950 elicits effective suppression of the inflammatory response at the cellular level.

## 3. Discussion

This study provides compelling evidence for the crucial role of the NLRP3 inflammasome in the pathogenesis of fat embolism (FE)-induced ARDS. Our findings demonstrate that FE leads to a significant upregulation of NLRP3 expression in the lungs, which correlates temporally with the development of pulmonary edema. Furthermore, administration of the NLRP3 antagonist, MCC950, effectively alleviated FE-induced pulmonary edema, lipid peroxidation, and histopathological lung damage through blockade of the ERK cascade. These observations demonstrate that the NLRP3 inflammasome serves as a key mediator of the inflammatory response and tissue injury associated with FE-induced ARDS.

The activation of the NLRP3 inflammasome is a well-established mechanism underlying various inflammatory conditions [5,6,7,19,20]. In the context of FE, both the presence of free fatty acids and the generation of reactive oxygen species (ROS) are critical triggers for NLRP3 inflammasome activation [5,11,21]. When fat globules accumulate within the pulmonary vasculature, they can induce cellular damage and oxidative stress, leading to the release of damage-associated molecular patterns (DAMPs) that activate the NLRP3 inflammasome [8,9,22]. The increased NLRP3 expression observed in our study at 4 **h** after FE coincides with the onset of pulmonary edema, suggesting a causal relationship between NLRP3 activation and the pathogenesis of FE-induced ARDS. Upon activation, the NLRP3 inflammasome promotes the maturation and secretion of the pro-inflammatory cytokine IL-1β, which is known to facilitate neutrophil recruitment and exacerbate alveolar and capillary epithelial damage [23,24].

Lungs tissue is characterized by double vascularization; the bronchial vasculature, associated with the thoracic aorta, and the pulmonary system, which is part of the air/blood barrier of the lung tissue [25,26]. The accumulation of fat globules in the pulmonary vasculature disrupts the integrity of the vascular endothelium, leading to increased permeability and subsequent development of pulmonary edema. Vascular endothelial growth factor (VEGF), a potent angiogenic factor, plays a crucial role in regulating vascular permeability [27]. The role of VEGF remains a subject of ongoing debate. Some studies suggest that the administration of PRIP-VEGF, which prevents the proteolytic degradation of VEGF, may play distinct yet pivotal roles in mediating inflammation in ARDS, representing a potential novel therapeutic approach for acute and chronic inflammatory pulmonary disease [28]. However, our previous study indicated that VEGF expression significantly increases after FE, which is mediated by ERK phosphorylation. It is also evidenced that NLRP3 activation is associated with VEGF overexpression, which further exacerbates the disruption of the vascular barrier and contributes to the pathogenesis of FE-induced ARDS [28,29]. In this study, we found the administration of MCC950 not only blocked the expression of NLRP3 but also inhibited ERK and Raf phosphorylation. However, the effects of MCC950 on VEGF expression after FE remain to be further verified, representing a limitation of this study. Nonetheless, this study suggests that MCC950 elicits dual-target effects by addressing both NLRP3 inflammasome activation and MAPK cascade activation, which induce vascular dysfunction. MCC950 may therefore offer a more comprehensive therapeutic strategy for managing FE-induced ARDS.

The alveolar epithelium consists of two main cell types: alveolar type I (AT I) and alveolar type II (AT II). AT I cells are complex, branched cells that provide the gas exchange surface in the alveolus, while AT II cells not only secrete surfactant but also repair damaged alveolar epithelium by dividing and acting as progenitor cells for both cell types [30]. Our immunofluorescence studies revealed that NLRP3 expression is predominantly localized in AT I cells, suggesting these cells serve as primary sites for NLRP3 activation and subsequent inflammatory signaling in FE-induced ARDS [31]. In addition, NLRP3 activation in AT II cells has been linked to pulmonary fibrosis by promoting myofibroblast differentiation from lung-resident mesenchymal stem cells [32]. Studies suggest that approximately 20% to 50% of patients who survive ARDS may develop some degree of pulmonary fibrosis, with the risk being higher in those with more severe syndromes. This risk is particularly significant in patients with COVID-19-related ARDS, among whom up to 60% may develop pulmonary fibrosis [33,34]. Although our findings show that NLRP3 is mainly expressed in AT I cells, we cannot exclude the role of AT II cells in FE-induced ARDS. We propose that FE-induced ARDS is a progressive clinical syndrome; at the early stage, AT I cells are activated first, interfering with pulmonary function. However, the effects of FE-induced NLRP3 activation in AT II cells, which may induce a more severe syndrome and contribute to pulmonary fibrosis, remain to be further verified. Therefore, blockade of NLRP3 activation in earlier phases is critical to protect animals against FE-induced pulmonary fibrosis.

Our findings show that MCC950 administration effectively reduces NLRP3 expression, pulmonary edema, IL-1β overexpression, lipid peroxidation, and histopathological damage in FE-induced ARDS. These results align with previous studies demonstrating the protective effects of NLRP3 inhibition in other forms of ARDS, including those induced by mechanical ventilation, sepsis, and viral infection [16,35]. Dysregulated activation of the NLRP3 inflammasome has been implicated in a range of diseases, including metabolic disorders and neurodegenerative diseases [36,37,38]. Various strategies exist for inhibiting NLRP3 activation, including indirect methods targeting NF-kB and IL-1β, and direct methods targeting NLRP3 itself. The latter provides higher efficacy and improved safety. MCC950, a diaryl sulfonylurea compound, directly binds to the Walker B motif within the NACHT domain of NLRP3 [39,40], preventing ATP hydrolysis and eliciting potent inhibition of NLRP3. Concerns have been raised regarding potential adverse renal effects of MCC950 in diabetic mice [40]. In their study, Østergaard et al. investigated both the short-term and long-term effects of MCC950 on diabetic mice. For short-term treatment, MCC950 administration began after 5 weeks of diabetes and continued for the remaining 5 weeks, resulting in a total diabetes duration of 10 weeks. For long-term treatment, MCC950 administration started after 9 weeks of diabetes and continued for another 9 weeks, with a total diabetes duration of 18 weeks. The dosage used in Østergaard et al.’s research was significantly higher than that used in the present investigation. In our pilot study, we tested three doses of MCC950 to evaluate its effects on FE-induced NLRP3 expression. The 5 mg/kg dose showed no inhibitory effect, whereas the 10 mg/kg and 20 mg/kg doses significantly reduced NLRP3 expression. We administered a single dose of 10 mg/kg, which demonstrated protective effects without any apparent toxicity, consistent with findings from previous studies [41]. Several other NLRP3 inhibitors, such as GDC-2394 [42,43], RRx-001 [44,45], CY-09 [46], and fluoxetine [47,48] have entered clinical trials to evaluate their safety and efficacy. The development of NLRP3 inhibitors may provide therapeutic strategies for the fat embolism syndrome. While we demonstrated MCC950′s preventive efficacy, its effectiveness in established injury and long-term consequences requires further investigation. Future research should focus on optimizing treatment timing and exploring combination therapies, particularly those targeting both NLRP3 and VEGF pathways, as these may provide synergistic protective effects.

**Figure 6:**
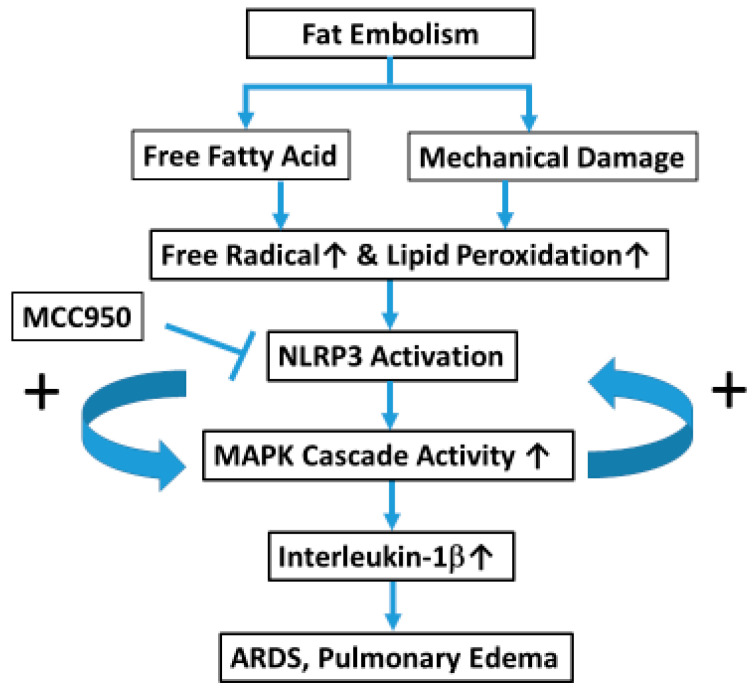
Proposed mechanisms of NLRP3 in ARDS.

In conclusion, our study provides compelling evidence that the NLRP3 inflammasome plays a central role in the pathogenesis of FE-induced ARDS. Therapeutic targeting of NLRP3 with MCC950 significantly attenuated the inflammatory response and tissue damage, highlighting its potential as a promising strategy for treating this severe condition. The intricate interplay between NLRP3 and cellular responses during both the acute and chronic phases of injury underscores the need for further investigation to refine therapeutic approaches and prevent long-term pulmonary complications following FE. Below, we present the proposed mechanism (Figure 6).

## 4. Materials and Methods

### 4.1. Fat Embolism Induction

Male Sprague Dawley rats, weighing 350–370 g, were purchased from BioLASCO, Co., Ltd., Taipei, Taiwan) and housed individually in a temperature-controlled animal colony at 24 °C, with a normal 12 h:12 h light/dark cycle. Fat embolism (FE) was induced by injecting 0.2 mL of fatty micelles into the tail vein. These micelles were prepared by extracting animal oil from rat adipose tissue and mixing it with water (1:1). This procedure reliably induced FE and subsequent acute lung injury. To investigate NLRP3′s role in fat embolism (FE)-induced edema and assess the therapeutic potential of MCC950, a specific NLRP3 inhibitor, MCC950 (10 mg/kg) was administered immediately following FE induction. All procedures followed the National Institutes of Health Guide for Care and Use of Laboratory Animals, and all protocols were approved by the Institutional Animal Care and Use Committee (IACUC) at the National Chia-Yi University (IACUC Approval Number: 105031) [49].

### 4.2. Pulmonary Edema Evaluation

Lung weight gain ratio (LWG) served as an index of pulmonary edema and acute lung injury (ALI), with higher ratios indicating more severe damage. After FE induction, lungs were excised and weighed to obtain the initial lung weight (initial LW). Specimens were then dried in an oven at 120 °C for 48 h to get the final lung weight (final LW). The LWG was calculated using the following equation: LWG% = (final LW − initial LW)/initial LW × 100% [50].

### 4.3. Malondialdehyde (MDA Assay)

Pulmonary lipid peroxidation was evaluated by measuring malondialdehyde (MDA) level, using a protocol modified from a previous study [51]. Briefly, 100 μL of tissue supernatant was mixed with 200 μL of 10% trichloroacetic acid (TCA) and placed in an ice bath for 15 min to facilitate protein precipitation. The tissue mixture was then centrifuged at 2200× *g* for 15 min at 4 °C. Following centrifugation, 200 μL of the supernatant was added to an equal volume of 0.67% thiobarbituric acid (TBA) and heated in boiling water (100 °C) for 10 min for color development. For the standard curve, malondialdehyde (Thermo Fisher Scientific Inc., Waltham, MA, USA) was serially diluted to concentrations ranging from 3.125 to 100 nm, with a blank control. Each standard was processed identically to the samples. Subsequently, 80 μL of each sample was transferred to a 96-well plate in triplicate, and the absorbance was measured at 532 nm (U-1900, Hitachi, Minato City, Tokyo, Japan).

### 4.4. Western Blot Analysis

Lung tissues were rapidly thawed in 6 volumes of homogenizing buffer containing protease inhibitor cocktail (#78430, Thermo Fisher Scientific Inc., Waltham, MA, USA.) Samples were homogenized using a sonicator, and nuclear proteins were isolated by T-PER tissue protein extraction reagent (#78510, Thermo Fisher Scientific Inc., Waltham, MA, USA). Proteins were separated by 10% sodium dodecyl sulfate (SDS) gel electrophoresis and transferred to a polyvinylidene difluoride membrane. The membranes were incubated with primary antibodies against NLRP3, ERK, phosphorylated ERK, Raf, and phosphorylated Raf overnight at 4 °C, followed by HRP-conjugated secondary antibodies for 1 h at room temperature. Bound antibodies were visualized by enhanced chemiluminescence assay (ECL) (Bio Kit Biotechnology, Inc., Miaoli, Taiwan). Signals were analyzed using a Quantity One digital imaging system (Bio-Rad, Hercules, CA, USA). NLRP3 expression levels were normalized to α-tubulin, while phosphorylated ERK and phosphorylated Raf were normalized to ERK and Raf, respectively. Relative optical density was adjusted for the respective internal and sham control [49].

### 4.5. Enzyme-Linked Immunosorbent Assay (ELISA)

Following FE induction or drug administration, animals were euthanized, and their lungs were collected, weighed, and rapidly thawed in 6 volumes of ice-cold homogenizing buffer. Samples were homogenized using a sonicator. Interleukin-1β (IL-1β) levels in pulmonary tissue were determined by ELISA (Abcam, Cambridge, UK) according to the manufacturer’s instructions [49].

### 4.6. Immunocytochemical Staining

After FE induction and MCC950 administration, animals were euthanized with pentobarbital (100 mg/kg, intraperitoneally) and perfused transcardially with 0.9% NaCl and 10% formalin. Pulmonary tissues were collected, fixed in 4% paraformaldehyde for 6 h, and immersed in 25% sucrose for 3–4 days at 4 °C. Tissues were then embedded and sectioned into 20-mm-thick slices using a cryostat. To minimize background staining, sections were pre-incubated in PBS containing 10% normal goat serum at room temperature. Double-labeling was performed using antibodies against NLRP3 and alveolar type I (AT I) cell marker aquaporin-5. Primary antibodies included mouse anti-NLRP3 (1:1000 dilution; Santa Cruz Biotechnology, Santa Cruz, CA, USA) and goat anti-aquaporin 5 (1:1000 dilution; Santa Cruz Biotechnology). Secondary antibodies were AlexaFluor 555 goat anti-mouse IgG (1:3000 dilution, Invitrogen, Carlsbad, CA, USA) and AlexaFluor 488 donkey anti-goat IgG (1:1000 dilution; Invitrogen). Images were captured using a Nikon Eclipse 80i fluorescence microscope (Nikon, Shinagawa City, Tokyo, Japan).

### 4.7. Hematoxylin and Eosin Staining

Following FE induction and MCC950 administration, animals were euthanized with pentobarbital (100 mg/kg, intraperitoneally) and perfused transcardially with 0.9% NaCl followed by 10% formalin. Pulmonary tissues were collected and embedded in paraffin blocks. Sections (5 µm thickness) were stained with hematoxylin and eosin (H&E) and examined microscopically. Pulmonary damage was assessed by qualifying immune cell infiltration [49].

### 4.8. Statistical Analysis

All data, including protein levels from immunoblotting, are presented as mean + standard error of the mean (SEM). Statistical analyses were performed by either a nonparametric Mann–Whitney U test or an analysis of variance (ANOVA) with Bonferroni–Dunn post hoc. A *p*-value < 0.05 was considered statistically significant [49].

## Data Availability

The data presented in this study are available on request from the corresponding author.
